# Temporary ICUs during the COVID-19 pandemic first wave: description of the cohort at a French centre

**DOI:** 10.1186/s12871-022-01845-9

**Published:** 2022-10-03

**Authors:** Nathalie Zappella, Chadi Dirani, Brice Lortat Jacob, Sébastien Tanaka, Elie Kantor, Adnan El Kalai, Yassine Rkik, Aurélie Gouel Cheron, Alexy Tran Dinh, Philippe Montravers

**Affiliations:** 1 Anaesthesiology and Critical Care Medicine Department, DMU PARABOL, Bichat – Claude Bernard Hospital, HUPNVS, AP-HP, Paris, France; 2 Paris University, Paris, France; 3 Antibody in Therapy and Pathology, Pasteur Institute, UMR 1222 INSERM, Paris, France; 4 Biostatistics Research Branch, Division of Clinical Research, National Institute of Allergy and Infectious Diseases, National Institutes of Health, Bethesda, MD USA; 5 Réunion Island University, French Institute of Health and Medical Research (INSERM), U1188 Diabetes atherothrombosis Réunion Indian Ocean (DéTROI), CYROI Platform, Saint-Denis, de La Réunion France; 6 INSERM UMR 1152, Paris, France

**Keywords:** COVID-19, ARDS, Intensive care, Sanitary crisis, Temporary ICU

## Abstract

**Background:**

During the COVID-19 first wave in France, the capacity of intensive care unit (ICU) beds almost doubled, mainly because of the opening of temporary ICUs with staff and equipment from anaesthesia.

**Objectives:**

We aim to investigate if the initial management in temporary ICU is associated with a change in ICU mortality and short-term prognosis.

**Design:**

Retrospective single-centre cohort study.

**Setting:**

Surgical ICU of the Bichat Claude Bernard University Hospital during the COVID-19 “first wave” (from 18 March to 10 April 2020).

**Patients:**

All consecutive patients older than 18 years of age with laboratory-confirmed SARS-CoV-2 infection and/or typical radiological patterns were included during their first stay in the ICU for COVID-19.

**Intervention:**

Patients were admitted to a temporary ICU if no room was available in the classical ICU and if they needed invasive mechanical ventilation but no renal replacement therapy or Extracorporeal Membrane Oxygenation (ECMO) in the short term. The temporary ICUs were managed by mixed teams (from the ICU and anaesthesiology departments) following a common protocol and staff meetings.

**Main outcome measure:**

ICU mortality

**Results:**

Among the 59 patients admitted, 37 (62.7%) patients had initial management in the temporary ICU. They had the same characteristics on admission and the same medical management as patients admitted to the classical ICU. ICU mortality was similar in the 2 groups (32.4% in temporary ICUs versus 40.9% in classical ICUs; p=0.58). SAPS-II and ECMO use were associated with mortality in multivariate analysis but not admission to the temporary ICU.

**Conclusion:**

In an overload context of the ICU of a geographical area, our temporary ICU model allowed access to intensive care for all patients requiring it without endangering them.

## Background

During the first coronavirus disease 2019 (COVID-19) wave (March-May 2020), intensive care units (ICU) in many areas of Europe were overwhelmed by patients developing severe hypoxemic pneumonia and acute respiratory distress syndrome (ARDS) [1–3]. To manage this major public health problem, various countries opened temporary hospitals by transforming exhibition centres: IFEMA (Institución Ferial de Madrid) in Madrid, Spain, Fiera in Milano, Italy, Corona Hospital in Berlin, Germany, and Nightingale Hospitals in several cities in the United Kingdom [4–6]. Similar structures were not developed in France. However, at the peak of the first wave, on April 8th, 4,806 ICU beds had been created, representing a 95% increase in ICU inpatient capacities [7]. These beds were opened by upgrading the acute care unit (45%), postanaesthetic care unit and operating theatre (35%), or conventional wards (10%). Only 10% of new beds were in new ICUs (10%) [7].

French authorities activated the national crisis plan (Plan Blanc) [8] to postpone all nonemergency surgery and medical procedures to increase available staff and equipment, mainly from post anaesthesia care units and operating rooms. Thus, in temporary ICUs, the staff comprised primarily anaesthesiologists [7]. In France, anaesthesiologists have a minimum of two years of ICU training during their curriculum and are one of the medical specialties allowed to work in the ICU [9]. However, most of them have no regular activity in the ICU.

This exceptional situation and numerous organizational changes required to create temporary ICUs have led to questions concerning the quality of the care provided. This issue has been minimally addressed in the literature to date, but some studies suggest that ICU overflow or a high rate of occupancy of beds compatible with invasive mechanical ventilation are associated with ICU mortality [10, 11].

This study aimed to evaluate whether the initial management of patients admitted for COVID-19 in the temporary ICU was associated with a modification of the short-term prognosis.

## Patients and methods

### Design of the study

This retrospective monocentric study was conducted on a cohort of patients admitted to the surgical ICU for COVID-19 during the “first wave” at the Bichat-Claude Bernard University Hospital in Paris (from March 18^th^ to April 10^th^, 2020).

### Ethics

Ethical approval for this study (IRB number 00010254-2020–236) was provided by the French Institutional Review Board (Comité d’Éthique de la Recherche en Anesthésie-Réanimation, Société Française d’Anesthésie Réanimation, 74 Rue Raynouard, 75016 Paris, France (Chairperson Prof J.E. Bazin) on 21 December 2020. This committee waived the need for signed informed consent. The French law (Law n°2012-300, March 5, 2012) mentions that no specific permission or license is required for observational retrospective studies. This was the case for the current investigation which did not modify the physician’s management of the patients.

### Patients

All consecutive patients older than 18 years of age with laboratory-confirmed SARS-CoV-2 infection and/or typical radiological patterns were included during their first stay in the intensive care unit for COVID-19. Lung transplant patients and postoperative patients for whom a therapeutic limitation had previously been decided were excluded from this analysis.

Two groups of patients were considered depending where they were admitted in: classical ICU or temporary ICU

**Fig. 1:**
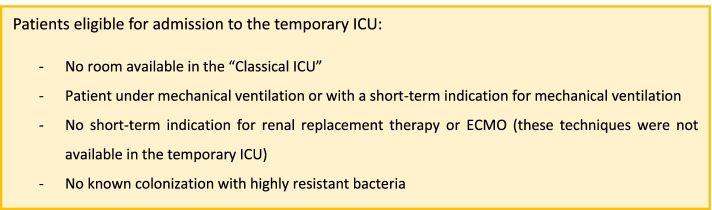
Criteria of admission to the temporary ICU

Patients admitted to the temporary ICU were selected according to our predefined criteria (Fig. 1). The rule was to provide any available classical ICU bed to the most severe patient of the temporary ICU. Similarly, when a high-performance ICU ventilator became available, it was attributed to the patient who needed it the most.

### Medical devices and architectural considerations

Temporary ICUs were opened in the recovery room (12 beds) and in 2 operating theatres (4 beds in each room). Therefore, all the temporary ICU beds were opened in common rooms. The beds were not ICU beds but standard beds. The respirators were ICU ventilators (Dräger Evita 4®) and anaesthesia ventilators (Dräger Primus® and Perseus®). Cushions for ventral decubitus installation were ordered in emergency. Renal replacement therapy (RRT) could not be performed in the temporary ICU because no specific water circuit was available and no nurse was trained on this technique. ECMO could be started in temporary ICUs with a subsequent transfer to the classical ICU for care continuation because of a lack of space between beds in temporary ICUs and because nurses were not trained in this technique.

The classical ICU comprised 8 ICU beds and 6 continuous care beds upgraded to ICU beds dedicated to COVID-19-infected patients (while maintaining 4 ICU beds for non-COVID-19 patients). This upgrading did not imply any structural modification but only modified the patient/nurse ratio. The ventilators used were Dräger Evita V500®, Dräger Evita XL®, Dräger Evita 4®, Dräger Evita 2 dura®, Dräger Savina®, and General Electric Carescape R860®.

At the peak of the crisis, 34 ICU beds for COVID-19 patients were opened in our service. We kept 4 ICU beds for non-COVID-19 patients during the entire period.

National health authorities organized ICU patients by interregional transfer to increase available beds in overcrowded areas [12]. Our service participated in sending patients accordingly.

### Medical and paramedical organization

The organization of the service during the crisis was as follows:

- A senior intensive care physician supervised each sector (comprising 6 to 12 beds) supported by a team of physicians comprising senior intensive care physicians from other sectors (anaesthesia, cardiac surgery anaesthesia and ICU, additional staff from other regions or from private structures) and anaesthesia and intensive care residents from all listed sectors. The senior supervisor remained in the same area throughout the crisis. In summary, on a weekday, a senior intensive care physician took care of 6 to 12 patients, assisted by senior anaesthesiologists and / or residents who each took care of 3 patients.
- Duty lists for night shifts had to be reinforced: as usual, the senior and resident physicians of the ICU managed the classical ICU (18 beds), emergency calls and admissions regulation. The senior anaesthesiologist duty list was kept for surgical emergencies. The on-call senior anaesthesiologist duty list was transformed into a duty list for temporary ICUs in the recovery room (12 beds). A resident duty list was created for the temporary ICU in the recovery room. A senior duty list was created for the temporary ICU in the operating theatre (maximum 8 beds).
- On weekends, patients were managed by the duty teams, assisted by an additional senior intensive care physician during the day.
- Initial management was structured using a written protocol.
- Written transmissions were available on a secured cloud.
- A daily staff meeting was organized, comprising all the classical ICU senior physicians and where the teams from each sector came once at a time to shorten their presence and avoid crowded meetings. This staff ensured homogeneous care throughout the entire department and permitted senior ICU physicians to have a global vision of the patients and organize transfers from one sector to another if needed or if a room was available in the classical ICU.
- The nurse-patient ratio is regulated in ICU in France and cannot exceed 2.5 patients to 1 nurse. This ratio has been respected during the COVID-19 crisis.

### Data collected

The baseline information collected at ICU admission included age, sex, delay between symptom onset and hospital admission, delay between hospital and ICU admission, comorbidities (body mass index (BMI), active smoking, hypertension, diabetes, chronic renal insufficiency, and immunodeficiency), severity score (SOFA score and SAPS2 score), ventilator features (invasive mechanical ventilation (IMV), PEEP, FiO_2_, PaO_2_/FiO_2_ ratio, PaCO_2_), and standard laboratory data. The PaO_2_/FiO_2_ ratio was calculated only for patients with IMV. We recorded if the patient had chest computed tomography imaging (CT-scan), the time between admission in ICU and CT-scan and the degree of lung involvement on chest CT-scan [13]. We recorded the use of antibiotics, antiviral, and anti-inflammatory drugs. During the ICU stay, specific ARDS treatments were recorded (continuous neuromuscular blockers, prone position, ECMO), such as ventilator features and standard laboratory data at Day 2 and Day 7. ICU complications, organ dysfunctions and the acquisition of multiresistant bacteria [14] within the ICU stay were assessed, including the use of vasopressive therapy, acute kidney failure requiring renal replacement therapy, ventilator-associated pneumonia (VAP), and tracheotomy for difficult ventilatory weaning. Clinical data were collected prospectively, biological data were collected retrospectively. We have no missing data.

### Endpoints

ICU mortality was the main outcome. The secondary outcomes were the duration of ICU stay, duration of invasive mechanical ventilation, duration of hospital stay, and vital status at hospital discharge and Day 90.

### Statistics

Two groups were considered according to the first ICU to which they were admitted: classical and temporary ICU.

The results are presented as absolute values and percentages (qualitative variables) or medians and interquartile ranges (quantitative variables). Qualitative values were compared between survivors and deceased individuals using Fisher’s test, and quantitative variables were compared using the Wilcoxon test, when appropriate. We used a multivariate logistic regression model using mortality as the dependent variable. Pertinent variables with *p*<0.10 on univariate analysis were introduced as predictive factors to the complete-case model, and initial management in temporary ICUs was forced in the model. Logistic models were evaluated for discrimination with the area under the curve and calibration using the Hosmer–Lemeshow test. Statistical analyses were performed using R version 4.0.3 and SAS© 9.4 (SAS Institute, Cary, NC, USA). A p value of 0.05 was chosen *a priori*.

## Results

**Fig. 2:**
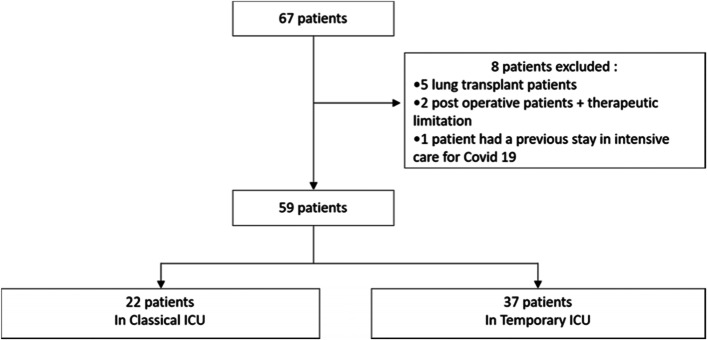
Flowchart of the study

From March 18 to April 10, 2020, 67 patients were admitted for respiratory failure due to COVID-19 in the ICU, of whom 59 met the inclusion criteria. Among them, 37 (63%) patients were first admitted to the temporary ICU. A flowchart is presented in Fig. 2.

**Fig. 3:**
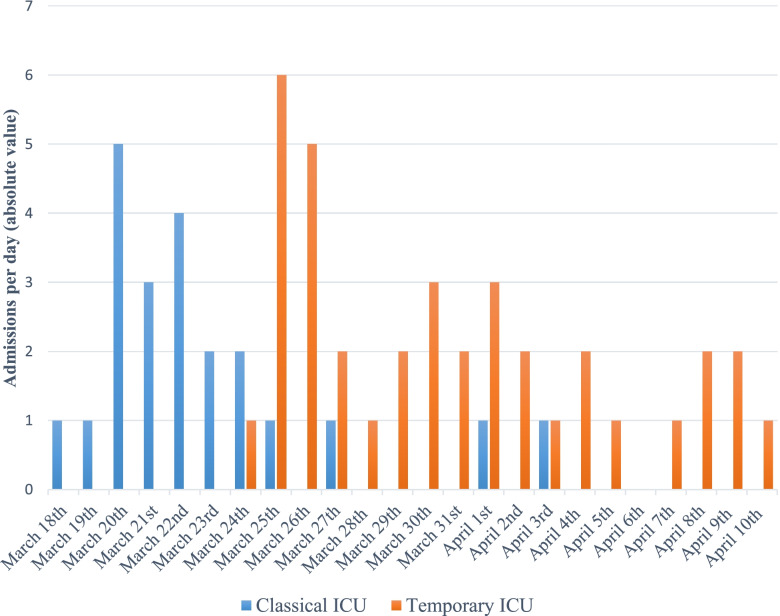
Distribution of admissions over time

The patient characteristics on admission (comorbidities, severity, and ventilator features) did not differ between the groups (Table 1). The distribution of admissions over time differed because the first patients were admitted to the classical ICU at the beginning of the crisis, while subsequent patients were admitted to the temporary ICU as beds were available (Fig. 3).

**Table 1:** Description of the cohort and comparison between patients initially managed in the classical ICU and patients initially managed in the temporary ICU

		All patients (*N* = 59)	Classical ICU (*N* = 22)	Temporary ICU (*N* = 37)	*P*
Epidemiological data					
Age, years, median [IQR]		56 [48-63.5]	53 [47.5-62.8]	57 [50-65]	0.58
Male sex, n (%)		41 (69.5)	17 (77.3)	11 (64.9)	0.48
Time between the first symptoms to hospital admission, days, median [IQR]		7 [4-10]	6 [3-9]	7 [5-10]	0.09
Time between the hospital and ICU admission, days, median [IQR]		1 [0-2]	0 [0-1]	1 [0-2]	0.15
Comorbidities					
Body Mass Index (kg/m2), median [IQR]		27.8 [ 24.6-31 ]	26 [24.4-29.6]	28.9 [24.9-31.1]	0.35
Hypertension, n (%)		30 (50.8)	12 (54.6)	18 (48.7)	0.34
Diabetes, n (%)		19 (32.2)	11 (50)	8 (21.6)	0.07
Chronic renal insufficiency, n (%)		4 (6.8)	2 (9.1)	2 (5.4)	0.16
Immunosuppression, n (%)		6 (10.2)	3 (13.6)	3 (8.1)	0.64
Active smoking, n (%)		6 (10.2)	2 (9.1)	4 (10.8)	0.71
Severity on admission					
SOFA score, median [IQR]		5 [4-7]	4.5 [3-6.8]	5 [4-7]	0.40
SAPS II score, median [IQR]		41 [30-53.5]	46.5 [26-54]	40 [31-53]	0.84
Computed tomographic data					
Chest CT-scan, n (%)		42 (71.1)	15 (68.2)	27 (73)	0.77
Chest CT-scan degree of lung involvement	Minimal < 25%	5 (11.9)	2 (13.3)	3 (11.1)	1
	Mild 25-50%	23 (54.8)	7 (46.7)	16 (59.3)	
	Moderate or severe > 50%	14 (33.3)	6 (40)	8 (29.6)	
Time between Chest CT-scan and ICU admission, days, median [IQR]		-0.6 [-2;0]	0 [-2;0]	-1 [-2;0]	0.40
Ventilator features on admission					
Invasive mechanical ventilation, n (%)		53 (90)	17 (77.3)	36 (97.3)	0.02
PEEP, cmH_2_O, median [IQR]		10 [10-11]	10 [10-11]	10 [10-10.8]	0.81
FiO2, %, median [IQR]		100 [70-100]	100 [60-100]	100 [70-100]	0.61
PaO2/FiO2, median [IQR]		130 [79.3-191.5]	160 [89-205]	122 [78.5-169]	0.42
Use of adjunct measures and outcome					
Continuous neuromuscular blockers, n (%)		48 (81.4)	15 (68.2)	33 (89.2)	0.08
Prone positioning, n (%)		41 (69.5)	13 (59.1)	28 (75.7)	0.24
ECMO, n (%)		11 (18.6)	6 (27.3)	5 (13.5)	0.30
Vasopressive therapy, n (%)		39 (66.1)	12 (54.5)	27 (73)	< 0.01
Renal replacement therapy for AKI, n (%)		11 (18.6)	4 (18.2)	7 (18.9)	1
Tracheotomy, n (%)		14 (23.7)	4 (18.2)	10 (27)	0.54
Interregional transfer		12 (20.3)	2 (9.1)	10 (27)	0.06
Ventilator-associated pneumonia, n (%)		30 (50.8)	8 (47)	22 (61.1)	0.11
Early ventilator-associated pneumonia, n (%)		4 (6.8)	1 (5.9)	3 (8.3)	1
Time between invasive ventilation and ventilator-associated pneumonia, days, median [IQR]		7 [5-10]	8 [5-10]	7 [5-11.5]	0.38
Duration of invasive ventilation, days	All patients, median [IQR]	14 [7.5–17.5]	9 [3.3-19.8]	16 [8-25]	0.07
	Survivors, median [IQR]	16 [5.3-27.3]	11[0-20]	20 [8-29]	0.10
ICU length of stay, days	All patients, median [IQR]	15 [8-28]	8.5 [3.8-19.8]	17 [9-28]	0.05
	Survivors, median [IQR]	19.5 [8.5-32]	12 [2-26]	26 [11-34]	0.11
ICU mortality rate, n (%)		21 (35.6)	9 (40.9)	12 (32.4)	0.58
Day-90 mortality rate, n (%)		21 (35.6)	9 (40.9)	12 (32.4)	0.58

Of the 37 patients admitted to the temporary ICU, 10 spent their entire stay in this sector, with an ICU LOS of seven [6.3-11] days. The other 27 patients were transferred to the classical ICU within four [2–8] days, with an overall ICU LOS of 26.5 [16-33] days.

No differences were found in medical management or patient outcomes (Table 1).

In univariate analysis, factors associated with mortality were the BMI, SAPS 2 on admission, PaO_2_/FiO_2_ ratio on admission, prone positioning, ECMO, the absence of chest-CT-scan and the absence of interregional transfer. Initial management in the temporary ICU was not associated with mortality (Table 2). Using multivariate analysis, only SAPS2 on admission and ECMO use were independently associated with ICU mortality (Table 3).

**Table 2:** Comparison between survivors and patients who died in the ICU

		Survivors (***N*** = 38)	Deceased in the ICU (***N*** = 21)	***p***
Epidemiological data				
Age, years, median [IQR]		54.5 [46.3-60.5]	59 [51-68]	0.11
Male sex, n (%)		27 (71)	14 (66.7)	0.77
Body Mass Index (kg/m2), median [IQR]		26.4 [23.4-29.4]	30.2 [26.4-35.4]	0.04
Severity on admission				
SOFA score, median [IQR]		4.5 [3-6]	5 [4-7]	0.11
SAPS II score, median [IQR]		35 [25.5-42]	51 [45-60]	< 0.01
Tomodensitometric data				
Chest CT-scan, n (%)		34 (89.5)	8 (38.1)	< 0.01
Chest CT degree of involvement	Minimal < 25%	4 (11.8)	1 (12.5)	0.55
	Mild 25-50%	20 (58.8)	3 (37.5)	
	Moderate or severe > 50%	10 (29.4)	4 (50)	
Ventilator features on admission				
Invasive mechanical ventilation, n (%)		32 (84)	21 (100)	0.08
PEEP, cmH_2_O, median [IQR]		10 [10-10]	10 [10-12]	0.10
FiO2, %, median [IQR]		100 [62.5-100]	100 [72.5-100]	0.50
PaO2/FiO2, median [IQR]		151.5 [108.8-230.2]	89.5 [66-146.5]	< 0.01
PaCO2, mmHg, median [IQR]		41 [38.5-42.3]	40 [35.3-43.8]	0.36
Drug therapies				
Antibiotic therapy, n (%)		37 (97.4)	20 (95.2)	1
Before ICU admission, n (%)		17 (44.7)	10 (47.6)	1
During ICU stay, n (%)		37 (97.4)	19 (90.5)	0.29
Antiviral therapy, n (%)		10 (26.3)	5 (23.8)	1
Hydroxychloroquine, n (%)		3 (7.9)	3 (14.3)	0.66
Remdesivir, n (%)		1 (2.6)	0	1
Lopinavir/ritonavir, n (%)		7 (18.4)	2 (9.5)	0.47
Other, n (%)		2 (5.3)	0	0.53
Anti-inflammatory treatment, n (%)		12 (31.6)	3 (14.3)	0.34
Dexamethasone, n (%)		7 (18.4)	2 (9.5)	0.47
Anti-IL-1 therapy, n (%)		1 (2.6)	1 (4.8)	1
Others, n (%)		5 (13.2)	1 (4.8)	0.41
Use of adjunct measures and outcome				
Continuous neuromuscular blockers, n (%)		29 (76.3)	19 (90.5)	0.30
Prone positioning, n (%)		22 (57.9)	19 (90.5)	0.02
ECMO, n (%)		2 (5.2)	9 (42.9)	< 0.01
Vasopressive therapy, n (%)		16 (42.1)	18 (85.7)	< 0.01
Renal replacement therapy for AKI, n (%)		5 (13.6)	6 (28.6)	0.17
Tracheotomy, n (%)		12 (31.6)	2 (9.5)	0.11
Ventilator-associated pneumonia, n (%)		19 (50)	11 (52.4)	1
Interregional transfer		11 (28.9)	1 (4.8)	0.04
Initial management in Temporary ICU		25 (65.8)	12 (57.1)	0.58

**Table 3:** Multivariate logistic regression of mortality in the whole cohort

	aOr	95% CI	***p***
Age	1.07	1.00-1.16	0.052
SAPS-II (per 5 points)	1.07	1.01-1.13	0.021
ECMO	27.2	3.64-369	0.004
Temporary ICU	1.37	0.32-6.67	0.7

No multiresistant bacterial acquisition was observed in either ICU.

## Discussion

To our best knowledge, this study is the first to describe and investigate the prognosis of patients managed in a temporary ICU. Beyond the medical value, this study demonstrates a specific episode of our recent sanitary history. It describes the adaptation of a French ICU team to a massive pandemic to increase ICU beds by threefold, without affecting patient care.

Patients initially admitted to the temporary ICU did not present an increased mortality rate compared with those initially admitted to the classical ICU.

Nevertheless, our results can roughly be generalized to all Temporary ICUs: there has been many different ways to manage Temporary ICUs. We have described here our way to make a temporary ICU work.

Ventilation strategies might have differed between ICU categories and, consequently, IMV duration, without sufficient power to relate this effect.

First, as the crisis evolved and the number of infected patients increased, the number of ICU beds decreased, and the demand for resuscitation beds increased. A shift in skills occurred with the implementation of noninvasive ventilation techniques and high-flow oxygen therapy in the classical ward. Although 27% of patients admitted to the classical ICU were not ventilated, only 3% of those admitted to the temporary ICU were not under IMV.

Second, ventilator availability in both ICUs differed. Ferré et al. used a transport ventilator without identifying consequences regarding the outcome [15]. Because of the lack of intensive care specialized ventilators at the beginning of the crisis, the temporary ICU in our department was equipped with either older model or anaesthesia ventilators. Our team has previously shown that anaesthesia ventilators can be used in these circumstances for 72-hour prolonged ventilation in ICU patients. However, in this preliminary observation, 2 of 16 COVID-19 patients required a ventilator shift for high plateau pressure or hypercapnia [16]. Other teams have encountered greater difficulties: Bottoroli reported increased mortality in COVID-19 patients exclusively ventilated with anaesthesia ventilators and reported several incidents, mostly airway obstruction and sudden ventilator failure [17]. This finding might be related to the fresh gas flow ranging from 80% of minute ventilation in patients receiving halogenates to 100% of minute ventilation in patients without inhaled aesthetics, while we used 150% of minute ventilation and no inhaled aesthetics [18]. In our cohort, no patient had been exclusively ventilated with anaesthesia machines. These devices were considered rescue devices, and they were replaced by ICU ventilators as soon as possible. In ARDS patients with difficult ventilation, less efficient ventilators may have made it more challenging to adjust ventilator parameters and monitor ventilation pressures and thus lung compliance. Consequently, we cannot exclude more frequent ventilator maladjustments and/or deeper sedation or even the use of neuromuscular blockers in some cases. The retrospective nature of our study did not allow us to collect data concerning respiratory mechanics, as plateau pressure was not systematically collected by the nurses.

Although the temporary ICUs were set in common rooms, we did not observe multiresistant bacterial transmission. The COVID-19 patient isolation protocol and strict adherence to hospital hygiene rules helped achieve this outcome.

Our cohort is small. To avoid biases, we have chosen to limit our observation to the first pandemic wave, only in our department. Indeed, the understanding of the disease and the practices have evolved over time. Similarly, the operating modes of the temporary ICU varied from one hospital to another.

We did not use noninvasive mechanical ventilation during the first wave because of the fear of an increased risk of contamination of healthcare workers without encouraging data in the literature at that time on acute hypoxemic respiratory failure [19].

Admission to temporary ICU was indicated for patients under IMV or with a short term indication of IMV. Nevertheless, as staging severity was difficult to assess with this new disease, we made some staging mistakes and admitted to temporary ICU patients who quickly improved and were not placed under IMV. This explains why a few patients without IMV were admitted to temporary ICU.

Chest CT-scans were performed before admission to the ICU. We note that patients who were going to die had less frequently a chest CT-scan, probably because they were considered too severe to tolerate this procedure before admission to the ICU. Also, at the time of the first wave, the degree of lung involvement was not yet assessed and did not help us to evaluate the severity of patients.

During the COVID-19 first wave, our team doubled its capacity to receive patients requiring intensive care because of the creation of temporary ICUs. In our cohort, 62.7% of patients were initially managed in temporary ICUs. The temporary ICU allowed access to intensive care for all patients requiring it immediately. This benefit could not be modelled in our study. However, during the first pandemic wave, on a national level, population access to ICUs was correlated with the global mortality observed [20, 21]. Similarly, the impact of resources shortening has been highlighted, particularly in elderly patients who were not considered a priority, given their poorer prognosis [22]. Beyond the care provided to each patient, ensuring universal access to diagnostics and treatment during such situations is crucial for a society to survive a health crisis [23].

The mortality observed in our cohort and associated risk factors are consistent with the main studies published during the related period [1, 2, 24, 25].

Temporary ICUs are an operating model developed in a health crisis context and should not represent a long-term strategy to increase regular ICU capacities. Although no loss of opportunity occurred related to managing patients presenting respiratory distress linked to SARS-CoV-2 infection during the first wave, this model certainly has collateral effects that are more difficult to measure.

We did not measure potential side effects that may have crucial consequences on the survivor’s life [26]: rate of delirium and posttraumatic stress [27] and occurrence of pressure sores or neurological lesions linked to the absence of specific beds or lower quality prone position material. This analysis would require a dedicated prospective study.

The potential psychological impacts of the development of temporary ICUs for all the actors in this particularly anxiety-provoking episode are not established: patients suffering from a new disease, patients ‘relatives who could not visit them, healthcare workers who had to work in unfamiliar surroundings

[28–31].

The development of temporary ICUs required surgical deprogramming, the consequences of which are difficult to assess at this time. In November 2020, the French hospital federation published the first global and national estimate of the impact of the epidemic on non-COVID-19 activity [32]. Compared to 2019, between March and August 2020, a 58% decrease was observed in total inpatient surgical activity and an 80% decrease in outpatient surgical activity, with significant temporal and geographical disparities, affecting both public and private structures.

The medicalization of these new units was ensured by the redeployment of 2,500 doctors, 80% of whom were anaesthesiologists, and 715 residents, 80% of whom were taking the anaesthesia and intensive care course. This unprecedented reinforcement clearly illustrates the value of a dual ability in anaesthesiology and intensive care.

## Conclusion

We did not observe increased mortality associated with initial management in the temporary ICU. This management in the temporary ICU, in the context of saturation of the classical ICU, managed by mixed teams made it possible to save lives without endangering these patients. The consequences in terms of morbidity can only be evaluated in larger groups.

## Data Availability

The datasets used and/or analysed during the current study are available from the corresponding author on reasonable request.

## Abbreviations

ARDS: Acute Respiratory Distress Syndrome
COVID-19: Coronavirus Disease 19
ECMO: Extracorporeal Membrane Oxygenation
ICU: Intensive Care Unit
IMV: Invasive Mechanical Ventilation
RRT: Renal Replacement Therapy
